# Intraoperative Use of High-Flow Nasal Cannula in Elderly Patients Undergoing Hip Fracture Repair Under Spinal Anesthesia: A Randomized Controlled Study

**DOI:** 10.7759/cureus.55846

**Published:** 2024-03-09

**Authors:** Majid F Mutar, Habiba Ben Hamada, Talib Razaq M Askar, Lassaad Hassini, Walid Naija, Mohamed Kahloul

**Affiliations:** 1 Department of Anesthesia and Intensive Care/Faculty of Medicine of Sousse, Sahloul Hospital/University of Sousse, Sousse, TUN; 2 Anesthesia Department, College of Medical Technology, Al-Ayen Iraqi University, Thi-Qar, Thi-Qar, IRQ; 3 Department of Anesthesia and Intensive Care/Faculty of Medicine of Souse, Sahloul Hospital/University of Sousse, Sousse, TUN; 4 Anesthesia Department, Faculty of Medicine, University of Thi-Qar, Thi-Qar, IRQ; 5 Department of Orthopedic Surgery, Faculty of Medicine of Sousse, Sahloul Hospital/University of Sousse, Sousse, TUN; 6 Department of Anesthesia and Intensive Care, Faculty of Medicine of Sousse, Sahloul Hospital/University of Sousse, Sousse, TUN

**Keywords:** noninvasive ventilation, high flow nasal cannula (hfnc), postoperative complication, geriatric surgery, elderly patient, hip fracture, anesthetic management

## Abstract

Background: The procedure of hip fracture repair poses a risk for postoperative pulmonary complications (PPCs) in elderly patients, accompanied by anesthesia and operations. Various noninvasive methods of respiratory support are used as prophylactic and therapeutic, mainly in the postoperative period.

Objective: This study aims to determine whether intraoperative use of a high-flow nasal cannula (HFNC) impacts elderly patient outcomes after hip fracture surgery.

Method: Seventy patients aged 65 and older undergoing traumatic hip surgery under spinal anesthesia for isolated hip fractures were randomly assigned to either an interventional group (I) utilizing a high-flow nasal cannula or a control group (C) without respiratory intervention in a six-month single-blind controlled study at Sahloul Teaching Hospital.

Results: The two groups had identical socio-demographic traits and baseline data. Respiratory postoperative complications occurred in two patients in group (I) and in nine patients in group (C), with a significant difference (p = 0.023). The main respiratory postoperative complications in group (I) were atelectasis (one case) and pulmonary edema (one case). The main respiratory postoperative complications in group (C) were atelectasis (four cases), pneumonia (two cases), COPD decompensation (two cases), and pulmonary edema (one case). No intensive care unit admissions or intraoperative complications were associated with using HFNC. The mean length of stay (LOS) in the hospital was 8.83 ± 2.91 for group I and 10.46 ± 3.4 for group (C), which differed significantly (p = 0.03) with no in-hospital mortality for the two groups.

Conclusion: The intraoperative administration of HFNC may lower the incidence of postoperative respiratory complications and the duration of hospital stays.

## Introduction

A high-flow nasal cannula (HFNC) is a new noninvasive technology for delivering warm (37 °C), humidified (100% relative humidity) oxygen at high flows with a reliable fraction of inspired oxygen (FiO_2_) of 21-100% or a combination of oxygen and air at a rapid flow exceeding 60 L/min. It can be delivered to the patient by the use of an air/oxygen blender, an active humidifier, a single heated tube, and a nasal cannula to support and improve oxygenation and lung ventilation [1]. Growing studies suggest that an HFNC as one of the present noninvasive oxygenation techniques could result in several clinical benefits by reducing inspiratory effort and breathing work, increasing end-expiratory volume and CO_2_ wash-out for the upper airways, and creating continuous positive airway pressure (CPAP) effects of 2-3 cmH_2_O in the upper airways. This CPAP effect, combined with an increase in CO_2_ wash-out and optimal airway humidification, could decrease the respiratory work of breathing and improve gas exchange [2]. In addition, it can meet the requirement for oxygen demand and deliver more accurate FiO_2_ [3]. HFNC can also induce optimal humidification, improving mucociliary function and patient comfort [1,4]. Even with a limited quantity of randomized clinical trials, HFNC has gained consideration as an alternative new respiratory support for critically ill patients. These effects assessed mainly in critical care patients may be extrapolated to the perioperative field, mainly in the intraoperative period.

The use of perioperative ventilatory support has been suggested as a prophylactic measure to reduce postoperative complications and enhance patients' recovery [5]. Noninvasive ventilation (NIV) is the most commonly used respiratory support perioperatively or in intensive care units. It can be used before anesthesia to promote pre-oxygenation before breathing through, particularly in obese individuals. Furthermore, it is also recommended in the management of PPC as it may prevent postoperative respiratory deterioration such as hypoxemia and acute respiratory failure (ARF), reduce the incidence of re-intubation, and decrease the hospital length of stay (LOS) [6]. Hip fracture repair for older patients is associated with adverse outcomes in terms of mortality, functional disability, and quality of life [7]. The one-year death rate following a hip fracture is approximately 20% [8]. Patients with frailty and underlying diseases are at risk of postoperative death along with significant morbidity due to several complications, including delirium, myocardial infarction, pneumonia, and cerebrovascular accidents [9]. Multiple studies have found that postoperative pulmonary complications (PPC) following significant operations are more prevalent than cardiac problems [10,11]. These PPCs have no clear definition. However, they usually refer to any postoperative aberration of the respiratory system that causes an identified illness or dysfunction, is potentially serious, and has a negative impact on patients' health [11,12]. This involves various complications such as atelectasis, bronchospasm, pneumonia, and respiratory failure [13]. Preoperative management to optimize these patients may enhance their outcomes [14]. The choice of the anesthetic approach is also because of its effects on perioperative hemodynamic status and postoperative rehabilitation quality. Local or regional pain relief is a viable option, but spinal anesthesia is among the most common [15].

This study seeks to evaluate the impact of intraoperative use of a high-flow nasal cannula on elderly patient outcomes after hip fracture surgery. We hypothesized that using HFNC with elderly patients undergoing hip fractures may have a positive impact on the patient’s health during and after surgery since this noninvasive technique has a direct effect on the physiology of the respiratory system. The primary aim was to determine the incidence of postoperative pulmonary complications as defined by the literature. The secondary study aims to evaluate the impact of intraoperative use of a high-flow nasal cannula on the length of hospital stay, in-hospital mortality, and patient satisfaction.

## Materials and methods

Study design

This randomized, controlled, single-blind study was implemented within six months in the orthopedic operating room of Sahloul Teaching Hospital in Sousse, Tunisia. After obtaining the ethics committee's approval of CHU Sahloul Sousse (HS 21-2023; Trial No.: PACTR202308575267647, Date of Approval: August 24, 2023, Trial Status: Registered in accordance with WHO and ICMJE standards) and patient written consent, 70 patients with isolated hip fractures aged over 65 years were scheduled and enrolled for traumatic hip surgery under spinal anesthesia. The sample size was calculated using G*Power, version 3.1.9.4 (Heinrich Heine University Düsseldorf, Düsseldorf, Germany). The calculation was power-based, using the following values: α = 0.15, power (1-β) = 0.85. Patients were randomly placed in two groups: the intervention group (I) and the control group (C). Group (I) was supplied with HFNC oxygen therapy (AIRVO™ 2; Fisher and Paykel Healthcare, Auckland, New Zealand) during surgery, which started immediately after the injection of spinal anesthesia and stopped at surgery completion. The oxygen flow rate and the fraction of inspired O2 were 35 l/min and 0.4, respectively. The control group (C) was not given any respiratory support or oxygen therapy during the entire surgery.

Randomization

Randomization was performed by an epidemiologist using Excel, version Professional Plus 2016 (Microsoft Corp., Redmond, WA, USA), employing a simple, parallel, double-blind randomization at a 1:1:1 ratio. Intervention group (I) with HFNC and control group (C) with no respiratory support device. The researcher, medical staff, and participants were not responsible for choosing which patient should get an HFNC device. When assigning study participants, each participant was given a private information form, numbered with the serial number assigned to the study type.

Intraoperative monitoring and anesthetic procedure

No premedication was administered before the operation. In the operating room, an 18-gauge intravenous cannula was placed in the cephalic vein, along with standard ECG leads, a pulse oximetry machine, and simple blood pressure (NIBP) monitoring. In the sitting position, an inter-spinous gap between L3/L4 was identified, and a 25-gauge pencil-point spinal needle was placed. After establishing the intrathecal position by monitoring the adequate flow of CSF, 2 ml of 0.5% hyperbaric bupivacaine and 2.5 μg sufentanil were administered intrathecally. Following spinal needle removal, a clean dressing was applied, and the patient was placed in a supine position. Depending on randomization criteria, HFNC was started in group intervention immediately after spinal anesthesia.

Data analysis

Collected data included sociodemographic characteristics (age, sex, body mass index, ASA score), fracture type (fracture of the neck of the femur, pertrochanteric fracture, subtrochanteric fracture), history of medical comorbidities, perioperative hemodynamic and respiratory parameters were monitored continuously during surgery and in the recovery room (respiratory rate [RR], heart rate [HR], arterial blood pressure [PA], peripheral arterial oxygenation [SPO_2_], intraoperative complications related to HFNC, patients outcomes; PPCs [e.g., atelectasis, bronchospasm, pneumonia, exacerbation of COPD], ICU admission, LOS, in-hospital mortality) and patient satisfaction assessed by a5 point Likert scale [16].

The primary outcome was the incidence of postoperative pulmonary complications. Secondary outcomes were hospital length of stay, patient in-hospital mortality, and patient satisfaction. 

The data were analyzed by IBM SPSS Statistics software (IBM Corp., Armonk, NY). Categorical variables were investigated by the Chi-square test, and numerical variables were compared by the t-student test. A p-value < 0.05 was considered statistically significant.

## Results

Regarding the stated aims and by following the study methodology, both groups were comparable regarding socio-demographic characteristics. The mean BMI was 25.44±4.12 in group I and 26.30±4.83 in group C (p = 0.66). The hip fracture was pertrochanteric in 31 patients in the intervention group and in 34 patients in the control group (p = 0.34) (Table 1).

**Table 1:** Comparison between groups regarding Socio-demographic characteristics and fracture type Group (I): interventional group (HFNC), Group (C): control group, P-value is considered significant when P<0.05.

Demographics	Group (I) N= 35	Group (C) N= 35	P-value
Age (mean)	77.77 ± 9.87	77.23 ± 8.9	0.81
Gender	0.52	0.84	0.32
BMI	25.44 ± 4.12	26.30 ± 4.83	0.66
ASA status: n (%)			
II	24 (68.57)	19 (54.28)	0.22
III	11 (31.42)	16 (45.71)	
Fracture type			
Pertrochanteric fracture	31 (88.57)	34 (97.14)	0.34
Neck of the femur fracture	3 (8.57)	1 (2.85)	
Subtrochanteric fracture	1 (2.85)	0	

The leading chronic conditions in the studied population were COPD/chronic bronchitis (n = 23), tobacco smoking (n = 25), and coronary diseases (n = 19), but without significant differences between groups (Table 2).

**Table 2:** Comparison between groups regarding chronic conditions

	Group I, n = 35, n (%)	Group C, n = 35, n (%)	p-Value
Diabetes	8	7	0.77
Hypertension	7	8	0.77
Congestive heart failure	3	5	0.45
Coronary diseases	8	11	0.42
Suspected sleep apnea	5	6	0.74
COPD/chronic bronchitis	11	12	0.79
Tobacco smoking	13	12	0.80

Regarding postoperative respiratory complications, a study showed that PPCs occurred in two patients in group (I) and in nine patients in group (C), with a significant difference (p = 0.023). Respiratory postoperative complications in group I were atelectasis (one case) and pulmonary edema (one case). The main respiratory postoperative complications in group C were atelectasis (three cases), pneumonia (two cases), COPD decompensation (two cases), and pulmonary edema (one case) (Table 3).

**Table 3:** Comparison between groups regarding postoperative pulmonary complications Group (I): interventional group (HFNC), Group (C): control group, PPC: postoperative pulmonary complications, COPD: chronic obstructive pulmonary disease

PPCs	Group (I) N = 35	Group (C) N = 35	P-value
PPC (all types) [n (%)]	2 (5.71)	9 (25.71)	0.023
Atelectasis [n (%)]	1 (2.85)	4 (17.14)	0.16
Pneumonia [n (%)]	0	2 (5.71)	0.15
COPD decompensation [n (%)]	0	2 (5.71)	0.15
Pulmonary edema [n (%)]	1 (2.85)	1 (2.85)	1

The mean length of hospital stay was 8.83±2.92 in the HFNC group and 10.49±3.39 in the control group, with a significant difference (p = 0.03). From both groups, no patient was admitted to intensive care units after surgery, and no patient developed intraoperative complications related to HFNC use. There was no in-hospital mortality in both groups. Satisfaction levels were comparable between both groups regarding the patient's, surgeon's, and anesthetist's satisfaction. However, patients were more satisfied in group C (p = 0.018) (Table 4).

**Table 4:** Satisfaction levels in patients

Patients satisfaction	Group I, n = 35	Group C, n = 35	P-value
Very satisfied	5	2	0.018
Somewhat satisfied	12	18	
Neither satisfied nor dissatisfied	4	8	
Somewhat dissatisfied	6	7	
Very dissatisfied	8	0	

Both groups were also comparable regarding baseline data, mainly respiratory rate (RR), oxygen saturation (SpO_2_), and hemodynamic status (arterial blood pressure and heart rate) (Table 3). The respiratory rate was considerably more significant in the HFNC group after 15 minutes (16.40±2.48 vs. 17.97±3.40; p = 0.03). Mean SpO_2_ was significantly higher in the intervention group at 15 minutes (98.45±0.97 vs. 94.57±2.52; p < 10^−^^3^), at 30 minutes (98.59±1.13 vs. 94.91± 2.74; p < 10^−3^), at 45 minutes (98.48±1.43 vs. 94.57±2.89; p < 10^−3^), at 60 minutes (98.57±1.43 vs. 95.06±2.49; p < 10^−3^), and at 90 minutes (98.20±2.94 vs. 95.07± 2.66; p = 0.45).

**Table 5:** Comparison between groups regarding initial hemodynamic and respiratory parameters Group (I): interventional group (HFNC), Group (C): control group, P-value: p-value is considered significant when (P<0.05). RR: respiratory rate, SpO_2_: saturation of peripheral oxygen, HR: heart rate, SAP: systolic arterial pressure, DAP: diastolic arterial pressure

Hemodynamic and respiratory parameters	Group (I), N = 35	Group (C), N = 35	P-value
RR	17.25 ± 2.44	16.94 ± 2.82	0.62
SpO_2_	95.11 ± 2.61	94.09 ± 2.92	0.12
HR	88.74 ± 17.65	95.54 ± 17.73	0.11
SAP	14.03 ± 1.87	14.2 ± 2.47	0.74
DAP	7.6 ± 1.11	7.8 ± 1.54	0.53

## Discussion

The study on HFNC application during traumatic hip surgery under spinal anesthesia in elderly patients with isolated hip fractures has offered significant information to confirm further respiratory support in the elder population context.

One of the strengths of this study is its precise design, which led to comparability in baseline socio-demographic characteristics, chronic conditions, and initial hemodynamic and respiratory parameters between HFNC intervention groups and control ones. This effect of randomization, evident from age-gender balance and BMI homogeneity, generates the findings' internal valid concentration. In a similar study, patients in both groups were carefully matched in terms of diagnosis and demographic characteristics. Hemodynamic and respiratory baseline parameters were similar in both cohorts. Intriguingly, the HFNC/AIRVO group demonstrated immediate post-extubation increases in pO_2_ and pO_2_/FiO_2_, but the CO_2_ washout did not significantly improve. Most notably, the frequency of reintubation and other ICU complications was comparable between groups [17].

Additionally, the rate of chronic comorbidity, including COPD/chronic bronchitis, tobacco smoking, and coronary heart diseases, was even between groups, which would reduce potential confounding impacts while further supporting the study design. Tobacco smoking, rather than COPD itself, is associated with the increased prevalence of cardiovascular comorbidities. On the other hand, those involved in dairy farming and exposed to organic dust have a relatively low prevalence of these comorbidities. This emphasizes the need to deal with and manage other established cardiovascular risk factors, even in mild-to-moderate cases of COPD [18].

Recently, HFNC has been reported as an effective measure for improving preoxygenation before intubation, mainly in difficult airway management and ICU cases. It may also enhance the patient's oxygenation after cardiac and thoracic procedures and reduce re-intubation risk [19]. Jin et al. reported similar findings in their study assessing the effects of postoperative use of HFNC on adult outcomes after major non-cardiothoracic and non-neurological surgeries [20]. Hemodynamic changes related to heart rate, which are significantly reduced in the HFNC group at different ventilation times, may be considered part of the reduced effort of breathing and in the same line with the reduced respiratory rate that was physiologically induced by HFNC and supported by patient satisfaction.

The HFNC group exhibited notable advantages in maintaining respiratory parameters throughout the surgical procedure, particularly achieving significantly higher oxygen saturation (SpO_2_) levels at various points in time. HFNC has also been used as respiratory support in sedated patients undergoing short interventions such as diagnostic and therapeutic bronchoscopy, with encouraging results [21-24].

Lee et al. used it in sedated patients undergoing endoscopic submucosal dissection with adequate oxygen saturation and patient satisfaction without significant respiratory events [25], as reported by Dong Liu et al. HFNC application during recovery from anesthesia reduces the postoperative time needed for waking up, agitation incidence, and increased lung function and oxygenation state [26]. These findings align with existing literature on HFNC, highlighting its efficacy in improving oxygenation and potentially contributing to reducing PPCs.

Despite these various uses of HFNC, its intraoperative use in awake patients has not yet been assessed. In this context, the study evaluated the efficacy and safety of intraoperative HFNC in patients with a higher risk of PPCs, mainly elderly patients undergoing hip fracture repair.

Supine position, stress of surgery, and aging are all factors that may decrease respiratory efficiency and increase the risk of PPCs. The physiological changes induced by HFNC can generate positive airway pressure, reduce dead space ventilation, improve carbon dioxide washing, increase tidal volume, decrease respiratory frequency and breathing work, and assist in recruiting lungs. In addition, it seems to be the most comfortable respiratory support, which may lead to better patient compliance.

During the surgical procedure, the HFNC group demonstrated notable superiority in maintaining respiratory parameters with exceptionally high SpO_2_ levels at different points. These data align with the currently available literature on HFNC, demonstrating its effectiveness for oxygenation and possibly reducing PPCs. In a case report, Wong et al. documented that intraoperative administration of high-flow nasal oxygen therapy resulted in satisfactory oxygenation, sustained PaCO_2_ at an acceptable level, and provided reasonable patient/surgeon satisfaction [27]. According to Yi et al., in patients undergoing awake craniotomies, HFNC was found safe and effective [28]. The same results were found in research enrolling 19 patients undergoing non-laser microlaryngoscopic surgery and managed intraoperatively with a flow nasal cannula. This safe and effective procedure spares patients the danger of consequences associated with intubation of the trachea and jet breathing while also clearing the surgical field vision [22].

The study's primary outcome measure, the incidence of PPCs, demonstrated a significant reduction in the HFNC group compared to the control group. This reduction was particularly notable for major respiratory postoperative complications, including atelectasis, pneumonia, COPD decompensation, and pulmonary edema. These results align with previous studies suggesting that HFNC positively influences respiratory outcomes, especially in surgical contexts. According to Taeil, HFNC is a safe, noninvasive oxygenation method that can effectively supply oxygen to pregnant patients with acute respiratory failure [29]. This intraoperative use in hypoxemic patients has also been reported in an elderly patient with pneumonia undergoing lower limb orthopedic surgery under spinal anesthesia. In this case, HFNC decreased the work of breathing and respiratory efforts, induced better patient comfort, and was associated with an excellent respiratory outcome [28]. Other studies evaluating the use of noninvasive positive pressure ventilation during surgery have had similar outcomes. The rationale behind patients' outcome improvement is the increased diaphragmatic excursion and total respiratory function [30]. However, patients' compliance seems to be better with HFNC.

In the current study, the implementation of HFNC may result in improved ventilation and oxygen saturation during surgery, a reduction in PPC incidence, and a shortened length of hospital stay. These promising results should be analyzed, considering some limitations. The definition of PPC was not consensual. In addition, as the more efficient exam in respiratory complication screening, the CT scan was impossible to perform in all cases. Finally, medium- and long-term outcomes were not assessed.

## Conclusions

A high-flow nasal cannula as a noninvasive respiratory support method guides us to hypothesize that if we apply it to elderly patients with major surgery (hip fracture surgery), it may reduce postoperative pulmonary complications. The authors concluded that HFNC might enhance patient oxygenation intraoperatively by increasing oxygen saturation and, as a result, decreasing the work of breathing, reducing the incidence of postoperative respiratory complications (atelectasis, pneumonia, COPD decompensation), increasing patient satisfaction, shortening the length of hospital stay, increasing the ICU admission rate, and reducing in-hospital mortality. The results confirm HFNC as an effective prophylactic therapy, especially in older, high-risk patients undergoing hip fracture repair with spinal block. A consensus could be confirmed with multiple clinical trials and a large population, which may support our results and conclusion.
